# Uterine infection-induced transcriptomic changes in the bovine endometrium

**DOI:** 10.3389/fvets.2026.1916650

**Published:** 2026-10-06

**Authors:** Mahfuz Rahman Adnan, Chin N. Lee, Vedbar S. Khadka, Birendra Mishra

**Affiliations:** 1 Department of Human Nutrition, Food and Animal Sciences, University of Hawaii at Manoa, Honolulu, HI, United States; 2 Department of Quantitative Health Sciences, University of Hawaii at Manoa, Honolulu, HI, United States

**Keywords:** bovine, infection, infertility, RNA-Seq, uteri

## Abstract

**Background:**

Uterine infections negatively impact cow health and productivity, increasing herd health costs, infertility, and culling. They also interfere with immune efficiency, sperm transport, embryonic development, and implantation, thus leading to pregnancy failure and infertility. Most previous studies have focused on transcriptomic changes induced by uterine infection in dairy cows. The present study aimed to investigate the changes in the endometrial transcript caused by uterine infections in beef cows.

**Methods:**

To achieve this, we examined the reproductive tract of over 200 Angus cows at a local slaughterhouse and collected intercaruncular endometrial tissues from a subset of infected cows (uteri filled with pus) and normal cyclic cows (*n* = 5/group). After isolating total RNA, the samples were subjected to high-throughput RNA sequencing (*n* = 5/group), and differentially expressed genes were validated using qPCR.

**Results:**

RNA seq revealed 954 differentially expressed genes (538 upregulated and 416 downregulated) in the bovine endometrium compared to infectious and normal uterine samples. The Ingenuity Pathway Analysis (IPA) showed 822 genes enriched in the bovine endometrium, with 530 upregulated and 292 downregulated in response to uterine infection. The activated immune system components include TLR receptors, NLR receptors, RLR receptors, cytokines, and chemokines. This enrichment of genes and molecules affects various biological processes and pathways, such as inflammatory and immune responses, cytokine-cytokine receptor interaction, and chemokine signaling.

**Conclusions:**

In summary, uterine infection alters the transcriptomic profile of the endometrium in infectious beef cows, potentially leading to pregnancy failure and infertility.

## Introduction

Uterine tract infection is a predisposing factor that affects the health and fertility of cows. These infections are also known as nonspecific infections and can affect tissues and organs other than the uterus (1). There are three main types of uterine infections: metritis, endometritis, and pyometra. If these infections occur in sequence, they can lead to infertility in cows (2). Uterine infections in cows can significantly impact herd health and productivity, leading to increased costs, reduced reproductive efficiency, culling, and the loss of valuable genetic heritage (1, 3). Several factors increase the risk of uterine infection, including difficult delivery, poor nutrition, inadequate hygiene during calving, retained fetal membranes, and various metabolic disorders (1). The healthy uterus is very resilient and maintains a clean environment throughout the reproductive cycle. This is possible due to strong physical and molecular barriers that protect the uterine lining from potential pathogens. These mechanisms are well-established and effective in maintaining a contamination-free environment. One risk factor for uterine infection is damage to the endometrial tissue, which can compromise the protective barriers and allow bacteria to enter. Additionally, during mating and childbirth, there is a risk of the uterine environment becoming contaminated by pathogens (4, 5). The immune system is usually activated and plays a significant role in maintaining uterine health. However, increased levels of progesterone have been found to suppress the immune system, making the uterus more vulnerable to infections. The risk of uterine infection is further heightened by reduced function of white blood cells, specifically neutrophils and lymphocytes (1, 6–8). Restoration of endometrial function and elimination of bacterial infections are facilitated by hematopoietic immune cells and the inflammatory response, which involves the secretion of cytokines and chemokines (5, 9). The innate immune response is crucial in preventing bacterial invasion (10–13). However, prolonged inflammatory responses can impair fertility by disrupting endocrine regulation, uterine hemostasis, and ovarian function (5). While uterine infections are common and can usually be treated easily, they can significantly impact uterine functions, which, in turn, can affect reproductive performance (14–17). Reported effects of clinical uterine infections in cows include longer estrus intervals, irregular ovarian cycles, postpartum luteal phases, delayed onset of ovarian cyclicity, and conception failure (5, 18).

For a successful pregnancy, a healthy uterine environment is necessary to support fetal growth and development (19). The bovine endometrium comprises luminal epithelial and underlying stromal cells, which are key in protecting against invading pathogens. Several bacterial species present in the uterus can cause increased inflammation, leading to the discharge of pus from the vagina. The infection caused by pathogenic agents adversely affects epithelial cells, leading to their disruption and the consequent exposure of stromal cells to toxins. This exposure, in turn, compromises immune efficiency, impeding sperm transport, embryonic development, and implantation. Three hypotheses exist regarding how uterine infections can impact fertility in cows. Firstly, an infection can disrupt endocrine signaling and the hypothalamic-pituitary-gonadal axis. Secondly, it may lead to ovarian dysregulation, reducing oocyte quality. Finally, it may impair the endometrium's ability to support embryonic development and implantation (5). Previous studies have examined changes in the endometrial transcriptome of dairy cows caused by uterine infections (5, 20–22). These studies have revealed that high-yielding dairy cows with negative energy balance and compromised immune systems are more susceptible to uterine infections. While monitoring the reproductive tracts of beef cows at a local slaughterhouse, several cases of uterine infections were observed. Despite normal ovarian morphology, including growing follicles and corpus luteum, the uteri were filled with pus. Based on these findings, the current study aims to investigate the transcriptomic changes in the endometrium of beef cows in response to uterine infections, using RNA-Sequencing (RNA-Seq) and Real-time quantitative PCR (qPCR) analyses.

## Materials and methods

### Sampling procedure

All animal experimentation and sampling were conducted in accordance with the guidelines approved by the Institutional Animal Care and Use Committee of the University of Hawai'i at Mānoa (Approval No. 18-3008). We examined the reproductive tracts of 200 Angus cows, aged between 3 and 5 years, at local abattoirs. Among these, nine cows were visibly healthy, with good body condition scores and no physical abnormalities. These cows exhibited normal ovarian morphology, as indicated by the presence of growing follicles and a corpus luteum. However, despite their visibly healthy appearance, these cows had pus-filled uteri and were classified as having infected uteri. For comparison, we collected uteri from cyclic cows that exhibited normal follicular waves and the presence of corpus luteum in their ovaries and had no pus in their uterine tracts, designated as cyclic cows. To investigate the endometrial transcriptomes, we collected intercaruncular endometrial tissues from a subset of infected cows (*n* = 5) and normal cyclic cows (*n* = 5), ensuring that the uteri were matched by corpus luteum stage (II) to avoid hormonal influences. The intercaruncular tissue regions were collected in well-labeled tubes containing RNAlater (Invitrogen, Thermo Fisher Scientific, USA) for transport. After that, RNA*later* was removed, and tissues were stored at−80 °C until analysis.

### RNA isolation and quality control

Total RNAs from the frozen intercaruncular tissues (60–100 mg) were isolated using TRIzol reagent (Invitrogen, Carlsbad, CA) following the manufacturer's instruction. RNAs concentration was determined using NanoDrop one (Thermo Fisher Scientific, Madison, WI), and the RNAs quality was determined by running on 2 % agarose gel. RNA quality was also analyzed using the Bioanalyzer (Agilent Technologies, Massy, France), and samples with an RNA integrity number (RIN) > 7 were subsequently used for RNA sequencing and quantitative real-time PCR. Total RNAs were stored at−80 °C for future use.

### Library preparation and sequencing

RNA-Seq libraries were prepared and sequenced at the Novogene Co., Ltd. (Beijing, China) facility. Briefly, 1 μg of RNA per sample was used as input material for RNA sample preparation. Following the manufacturer's recommendations, sequencing libraries were generated using NEBNext^®^ Ultra TM RNA Library Prep Kit for Illumina^®^ (NEB, USA), and index codes were added to attribute sequences to each sample. Briefly, mRNA was purified from total RNA using poly-T oligo-attached magnetic beads. Using divalent cations under elevated temperature, Fragmentation was carried out in NEBNext First Strand Synthesis Reaction Buffer (5X). First-strand cDNA was synthesized using a random hexamer primer and M-MuLV Reverse Transcriptase (RNase H-). Second-strand cDNA synthesis was performed using DNA polymerase I, and RNase H. Remaining overhangs were converted into blunt ends via exonuclease/polymerase activities. After adenylation of 3′ ends of DNA fragments, the NEBNext Adaptor with hairpin loop structure was ligated to prepare for hybridization. To select cDNA fragments of preferentially 150–200 bp in length, the library fragments were purified with the AMPure XP system (Beckman Coulter, Beverly, USA). Then, 3 μl USER Enzyme (NEB, USA) was used with the size-selected adaptor-ligated cDNA at 37 °C for 15 min, followed by 5 min at 95 °C before PCR. Then, PCR was performed with Phusion High-Fidelity DNA polymerase, Universal PCR primers, and Index (X) Primer. At last, PCR products were purified (AMPure XP system), and library quality was assessed on the Agilent Bioanalyzer 2,100 system.

According to the manufacturer's instructions, the index-coded samples were clustered on a cBot Cluster Generation System using the PE Cluster Kit cBot-HS (Illumina). After the cluster generation, the library preparations were sequenced on an Illumina platform, generating paired-end reads.

### RNA sequence analysis

RNA sequences were analyzed at the University of Hawaii John A. Burns School of Medicine Bioinformatics core (UHCC GBSR) as previously described (23). Paired-end reads in the FASTQ format were explored using FastQC (Babraham Institute, Cambridge, UK) and cleaned using Prinseq, a Perl script (24). The cleaning procedure included trimming low-quality reads from both 3′ and 5′ ends until a base pair of Phred quality score of 30 (99.9% accurate) or greater was found and filtering out reads with a mean quality score of less than 30 and length below 30 nucleotides. Cleaned reads were aligned against the bovine reference genome [Bos_taurus.ARS-UCD1.2(GCA_002263795.2)] using HiSAT2 (2.2.1). The resulting SAM files were sorted and converted to BAM files using SAMtools (1.12). Htseq-count script from the HTSeq (0.13.5) package generated read counts mapped to bovine gene models. Finally, differential gene expression analysis between the infectious and control groups was conducted using Bioconductor DESeq2 (1.32.0).

The identification of differentially expressed genes (DEGs) in the bovine endometrium was based on two criteria: genes that exhibited at greater/lesser than two-fold change (FC) and a Benjamini and Hochberg q-value of less than 0.05. The calculation of DEGs involved comparing infectious uteri with controls. Specifically, genes with higher expression levels were categorized as upregulated, whereas those with lower expression levels were classified as downregulated.

### Pathways analysis (pathways and network analysis)

#### Functional annotation and gene ontology enrichment analysis

To understand the Gene Ontology (GO) terms associated with upregulated and downregulated genes in bovine endometrium, we used the Database for Annotation, Visualization and Integrated Discovery (DAVID) system, an open web resource available at https://davidbioinformatics.nih.gov/. We uploaded the official gene symbols of the upregulated and downregulated genes to the functional annotation tool in the DAVID system and chose the bovine genome as the reference genome. The genes that matched the genes in DAVID were then classified into three GO terms: biological process (BP), cellular component (CC), and molecular function (MF). All GO terms were enriched with a modified *p*-value <0.05 and a threshold gene count of 2.

#### Kyoto encyclopedia of genes and genomes (KEGG)

To gain insight into KEGG pathways associated with the upregulated and downregulated genes in the bovine endometrium, the DAVID system was used. The functional annotation tool in the DAVID system was used to upload the official gene symbols of the upregulated and downregulated genes, and the bovine genome was selected as the reference genome. The enrichment parameters were set to a threshold gene count of 2 and a modified Fisher Exact *P*-value less than 0.05. The over-represented KEGG pathway terms were considered enriched KEGG pathways.

#### Ingenuity pathway analysis (IPA) and networks

Ingenuity Pathway Analysis (IPA) is a powerful human genome-based search tool with several advanced functions that enable insightful data analysis and interpretation. The differentially expressed genes (DEGs) were subjected to the IPA (QIAGENInc., https://www.qiagenbioinformatics.com/products/ingenuity-pathway-analysis) (25) to gain insights into canonical pathways and network discovery.

### Validation of RNA-Seq data using quantitative real-time PCR (qPCR)

The first strand of cDNA was synthesized from total RNA (20 μl reaction of RT mixture) using a High-Capacity cDNA Reverse Transcription Kit (Applied Biosystems, Foster City, CA). The cDNA was diluted 10-fold by adding 180 μl of nuclease-free water.

The qPCR assay was performed using a QuantStudio™ 3 System (Applied Biosystems, Foster City, CA) (50). Briefly, the qPCR reaction (10 μl) consisted of 3 μl cDNA, 1 μl each of forward and reverse primers (100μM) specific to the target gene, and 5 μl of PowerUp SYBR Green Master Mix (Applied Biosystems). The NCBI primer blast tool was used to design the forward and reverse primers for the selected genes (Supplementary Table 1). In a 96-well PCR plate, the PCR mixture and cDNA samples were loaded. The plate was sealed with clear adhesive film and briefly spun before being inserted into the QuantStudio™ 3 machine. The qPCR was programmed at 50 °C for 2 min (hold), 95 °C for 2 min (hold), followed by 40 repeat cycles of 95 °C for 15 s (denaturation), 60 °C for 15 s (annealing), and 72 °C for 1 min (extension). To confirm the sequence-specific PCR products, a melting curve was also generated. The specificity of the primers was also confirmed by running the PCR products on a gel electrophoresis. To select the most stable housekeeping gene in the endometrial tissues, three housekeeping genes, Glyceraldehyde 3-phosphate dehydrogenase (*GAPDH*), Beta-actin (β-actin), and TATA-Box Binding Protein (*TBP*), were run in triplicate. Beta-actin was the most stable and uniform gene across the samples. The selected genes (Supplementary Table 1) were run in triplicates across the samples, and the expression level was determined using cycle threshold (Ct) values after normalization with *Beta-actin*. The 2^−Δ*ΔCt*^ method was used to calculate the fold change for each gene. Data for fold change were presented as a mean ± standard error on the bar diagram.

### Statistical analysis

In RNA sequencing, genes with a fold change (FC) greater than 2 in the endometrial sample and a Benjamini and Hochberg q-value of less than 0.05 were identified as differentially expressed. The fold-change values for qPCR results were computed and analyzed using the 2^−Δ*ΔCt*^ method. The design of the study was completely random. The values were subjected to a student's t test to determine the significance. This was done using GraphPad prism (Version 8.0.2) while considering a P-value less than 0.05 for significant differences.

## Results

### Differential gene expression analysis

The transcriptomic profile of the uterine tract affected by infection was compared to that of normal uteri using RNA-seq. The RNA-seq libraries provided sequencing data in FASTQ format. On average, 92.71% of the retained reads from normal uteri and 92.11% from the infectious uteri were uniquely mapped to the bovine genome database using the Ensemble alignment of the bovine genome assembly. Analysis of the bovine genome assembly revealed 21,696 annotated gene transcripts. The differentially expressed genes (DEGs) had a log2 fold change greater than 2 and a *p*-value less than 0.05.

A total of 954 genes were annotated as differentially expressed between infectious and normal uterine samples (Figure 1A). Among these DEGs, 538 (56.4%) were upregulated, while 416 (43.6%) were downregulated in the infectious uteri compared to normal uteri. The top 20 upregulated and downregulated genes are presented in Tables 1, 2, respectively. A visual representation of the differentially expressed genes is shown in Figure 1B, while the distribution of the top 10 upregulated and downregulated genes across the uterine tract samples is illustrated in Figure 1C.

**Figure 1:**
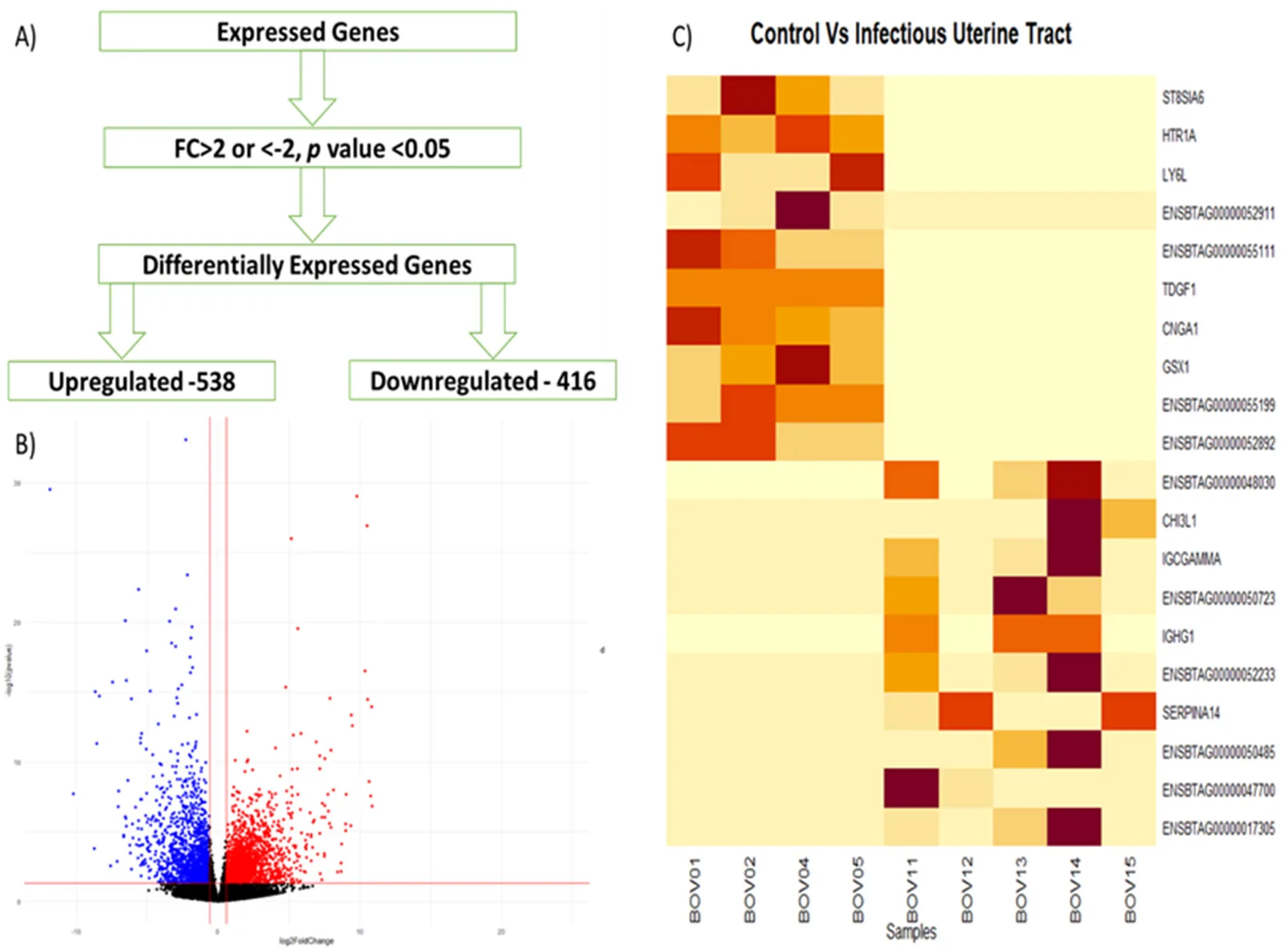
**(A)** Differentially expressed gene status, **(B)** Volcano plot showing the distribution of upregulated and downregulated genes, **(C)** Heatmap of top 10 upregulated and top 10 downregulated genes across the samples.

**Table 1:** The 20 most upregulated genes in bovine endometrium (Infectious vs. Normal).

Sl No	Gene ID	Gene description	Fold change (log2)
1	*ENSBTAG00000017305*	Uncharacterized protein (Protein: F1N160)	10.86
2	*ENSBTAG00000047700*	Uncharacterized protein (Protein: A0A3Q1LRW4)	10.83
3	*ENSBTAG00000050485*	Ig-like domain-containing protein (Protein: F1MLW7)	10.77
4	*SERPINA14*	Serpin peptidase inhibitor, clade A (alpha-1 antiproteinase, antitrypsin), member 14	10.66
5	*ENSBTAG00000052233*	Ig-like domain-containing protein (Protein: A0A3Q1N2B9)	10.54
6	*IGHG1*	Immunoglobulin heavy constant gamma 1	10.52
7	*ENSBTAG00000050723*	Uncharacterized protein	10.36
8	*IGCGAMMA*	Immunoglobulin heavy constant gamma 2	9.79
9	*CHI3L1*	Chitinase 3 like 1	9.48
10	*ENSBTAG00000048030*	Ig-like domain-containing protein (Protein: G3MZE0)	9.41
11	*C15H11orf34*	Chromosome 15 open reading frame, human C11orf34, also known as PLET1 (Placenta-expressed transcript 1 protein)	9.38
12	*ENSBTAG00000050373*	Ig-like domain-containing protein (Protein: A0A3Q1LSF0)	9.04
13	*DMBT1*	Deleted in Malignant Brain Tumors 1	8.99
14	*SLPI*	Secretory leukocyte peptidase inhibitor	8.99
15	*LOC112443200*	Immunoglobulin heavy variable 4-59-like	8.70
16	*BOLA-DQA5*	Major histocompatibility complex, class II, DQ alpha 5	8.68
17	*ENSBTAG00000050136*	Ig-like domain-containing protein (Protein: A0A3Q1MSF6)	8.15
18	*LOC100300716*	Immunoglobulin heavy variable 4-38-2	7.98
19	*CCL19*	C-C motif chemokine ligand 19	7.96
20	*ENSBTAG00000003408*	Uncharacterized protein (Protein: F1MH40)	7.89

**Table 2:** The 20 most downregulated genes in bovine endometrium (Infectious vs. Normal).

Sl No	Gene ID	Gene description	Fold change (log2)
1	*ENSBTAG00000052892*	Ig-like domain-containing protein (Protein: G3MZI4)	−11.85
2	*ENSBTAG00000055199*	–	−10.23
3	*GSX1*	GS homeobox 1	−8.73
4	*CNGA1*	Cyclic nucleotide-gated channel subunit alpha 1	−8.67
5	*TDGF1*	Teratocarcinoma–derived growth factor 1	−8.57
6	*ENSBTAG00000055111*	Ig–like domain–containing protein (Protein: A0A3Q1N268)	−8.37
7	*ENSBTAG00000052911*	Protein coding: Beta defensin (A0A3Q1MCB8)	−7.60
8	*LY6L*	Lymphocyte antigen 6 family member L	−7.43
9	*HTR1A*	5–hydroxytryptamine receptor 1A	−7.12
10	*ST8SIA6*	ST8 alpha–N–acetyl–neuraminide alpha−2,8–sialyltransferase 6	−7.04
11	*LOC112444733*	Collectin−46–like	−7.00
12	*LOC100847874*	Carbonic anhydrase 1–like	−6.65
13	*BPIFA1*	BPI fold containing family A member 1	−6.65
14	*PRKCG*	Protein kinase C gamma	−6.57
15	*ENSBTAG00000036102*	Ig-like domain-containing protein (Protein: G5E641)	−6.55
16	*PIPOX*	Pipecolic acid and sarcosine oxidase	−6.50
17	*CBLN4*	Cerebellin 4	−6.50
18	*TMIE*	Transmembrane inner ear	−6.48
19	*ENSBTAG00000048395*	Uncharacterized protein (Protein: A0A3Q1NCG5)	−6.36
20	*SH2D4B*	SH2 domain containing 4B	−6.23

### Gene ontology analysis

#### Upregulated differentially expressed genes

After identifying 538 upregulated DEGs, they were submitted to the DAVID system for functional annotation. The system annotated 425 genes and identified 342 genes involved in biological processes. These genes enriched several GO terms, including inflammatory and immune responses, with 29 and 26 genes, respectively. Additionally, the cellular component recognized 382 genes, with several enriched GO terms, including the nucleus and extracellular exosome, which had 83 and 65 genes, respectively. The molecular function recognized 319 genes, which enriched several functional pathways, including GTP binding, protein homodimerization, and chemokine activity. A visual representation of the upregulated DEGs enriching different gene ontology terms, including several pathways, is shown in Figure 2 and Supplementary Table 2.

**Figure 2:**
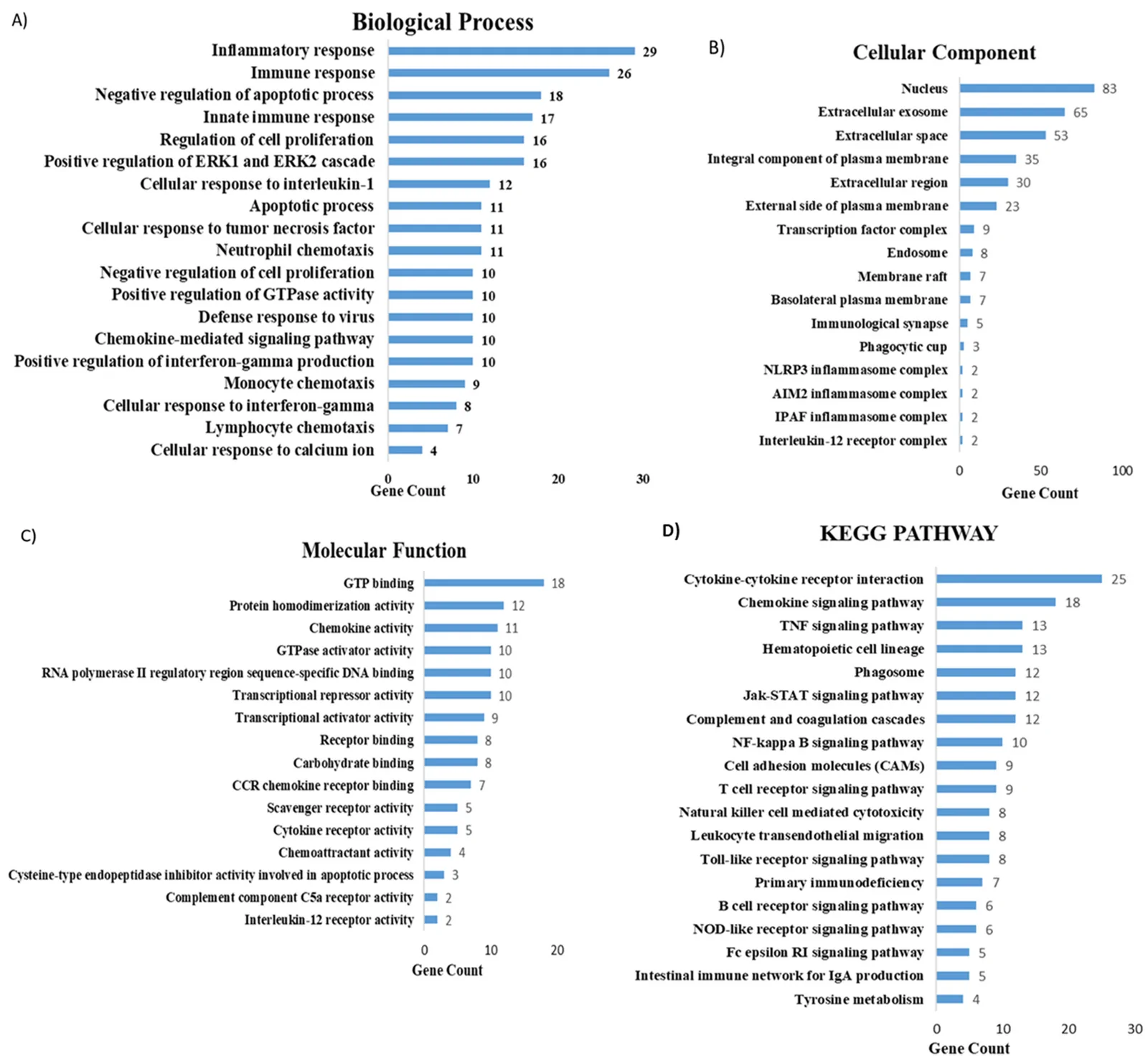
Gene Ontology enrichment analysis of the upregulated DEGs in the bovine endometrium; **(A)** Biological Process; **(B)** Cellular Component; **(C)** Molecular Function **(D)** KEGG pathway analysis of the upregulated DEGs in the bovine endometrium.

#### Downregulated differentially expressed genes

After identifying 416 downregulated DEGs, they were uploaded to the DAVID system for functional annotation. The system annotated 308 genes and recognized 234 genes involved in biological processes. Several GO terms (26) were enriched, with the oxidation-reduction process being the most significant, involving 11 genes. The cellular component analysis identified 265 genes, and among the enriched GO terms (12), the integral component of the membrane was recognized with 85 genes. The molecular function analysis identified 232 genes, which were enriched in several functional pathways (18), including heme-binding (9 genes), iron ion binding (8 genes), and monooxygenase activity (7 genes). Figure 3 and Supplementary Table 3 show a visual representation of the downregulated DEGs enriching different gene ontology terms and pathways.

**Figure 3:**
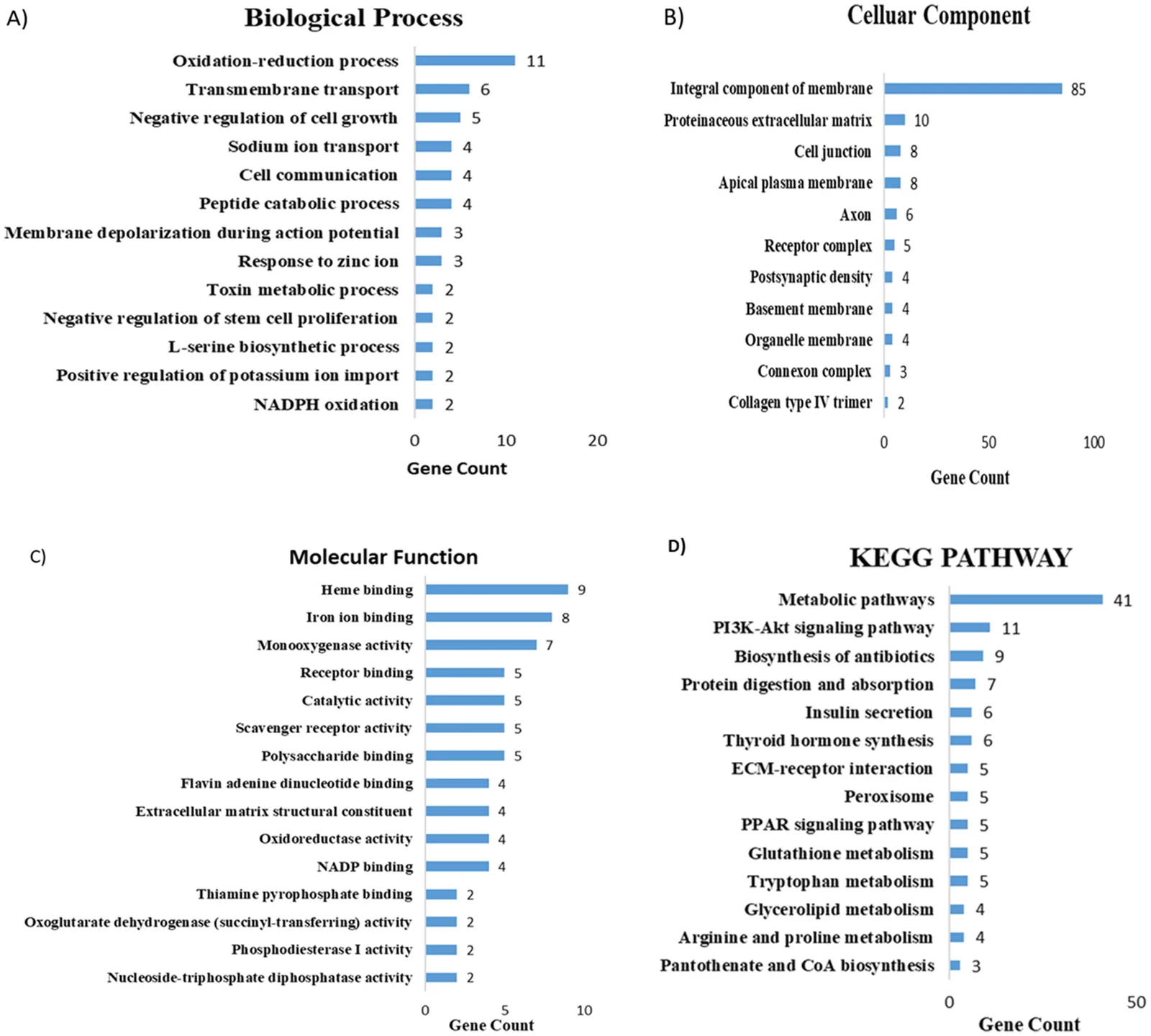
Gene Ontology enrichment analysis of the downregulated DEGs in the bovine endometrium; **(A)** Biological Process; **(B)** Cellular Component; **(C)** Molecular Function **(D)** KEGG pathway analysis of the downregulated DEGs in the bovine endometrium.

### KEGG pathway analysis

#### Upregulated KEGG pathways

In the DAVID system, 538 upregulated DEGs were uploaded for functional annotation. The system annotated 39 pathways, and 308 genes were identified within these pathways. Cytokine-cytokine receptor interaction and chemokine signaling pathways were among the top pathways, annotating 25 and 18 genes, respectively. Figure 2 and Supplementary Table 4 provide a visual representation of differentially expressed upregulated genes that enrich several KEGG pathways.

#### Downregulated KEGG pathways

In the DAVID system, 416 downregulated DEGs were uploaded for functional annotation. The system annotated 17 pathways that recognized 125 genes. The top two pathways were metabolic (41 genes) and PI3K-Akt signaling (11 genes). Figure 3 and Supplementary Table 5 present a visual representation of differentially expressed downregulated genes that enrich several KEGG pathways.

### Ingenuity pathway analysis

#### Canonical pathway

The DEGs were subjected to Ingenuity Pathway Analysis, which identified 822 molecules (Adj. *P*-value <0.05 and log2FoldChange > 2 or <-2). Among these molecules, 530 were upregulated, and 292 were downregulated, enriching 488 pathways. The top five canonical pathways, ranked by significance, were the Th2 Pathway, Granulocyte Adhesion and Diapedesis, Th1 and Th2 Activation Pathway, Tumor Microenvironment Pathway, and Role of Hypercytokinemia/hyperchemokinemia in the Pathogenesis of Influenza (Figure 4A). Out of the 488 pathways, 117 were predicted to be activated, 32 were predicted to be inhibited, and 321 had no discernible activity pattern. The remaining pathways were predicted to be neither activated nor deactivated (Figure 4B).

**Figure 4:**
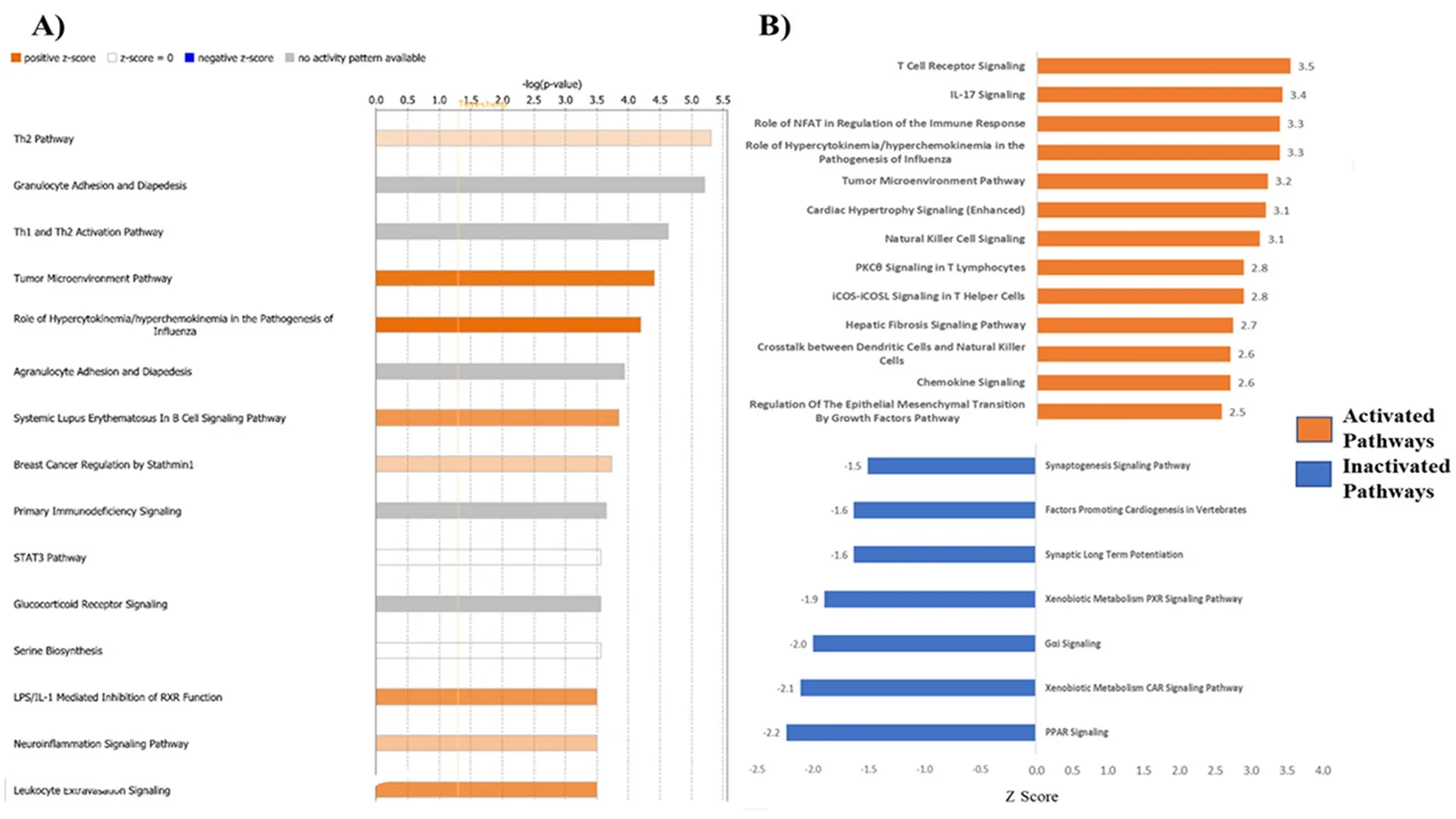
Top significant ingenuity canonical pathways. **(A)** Top canonical pathways. **(B)** Top predicted canonical pathways.

#### IPA networks

The IPA analysis revealed 25 enriched networks of differentially expressed molecules and their associated diseases and functions. The top 10 networks with the focus molecules are presented in Figure 5. These networks showcase the diverse roles of the molecules in regulating the activity, inhibition, and expression patterns of various proteins at different levels (Figure 6).

**Figure 5:**
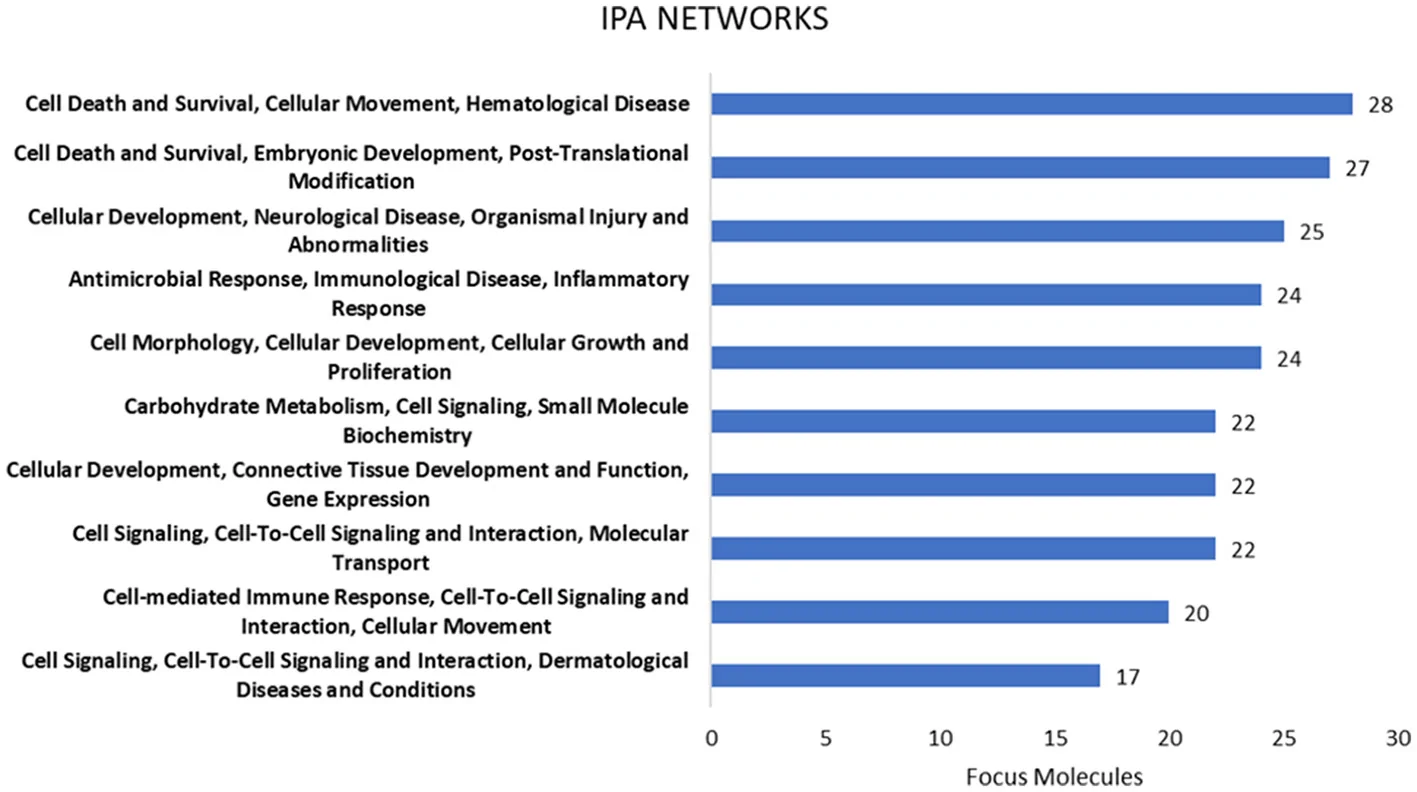
Top 10 IPA networks.

**Figure 6:**
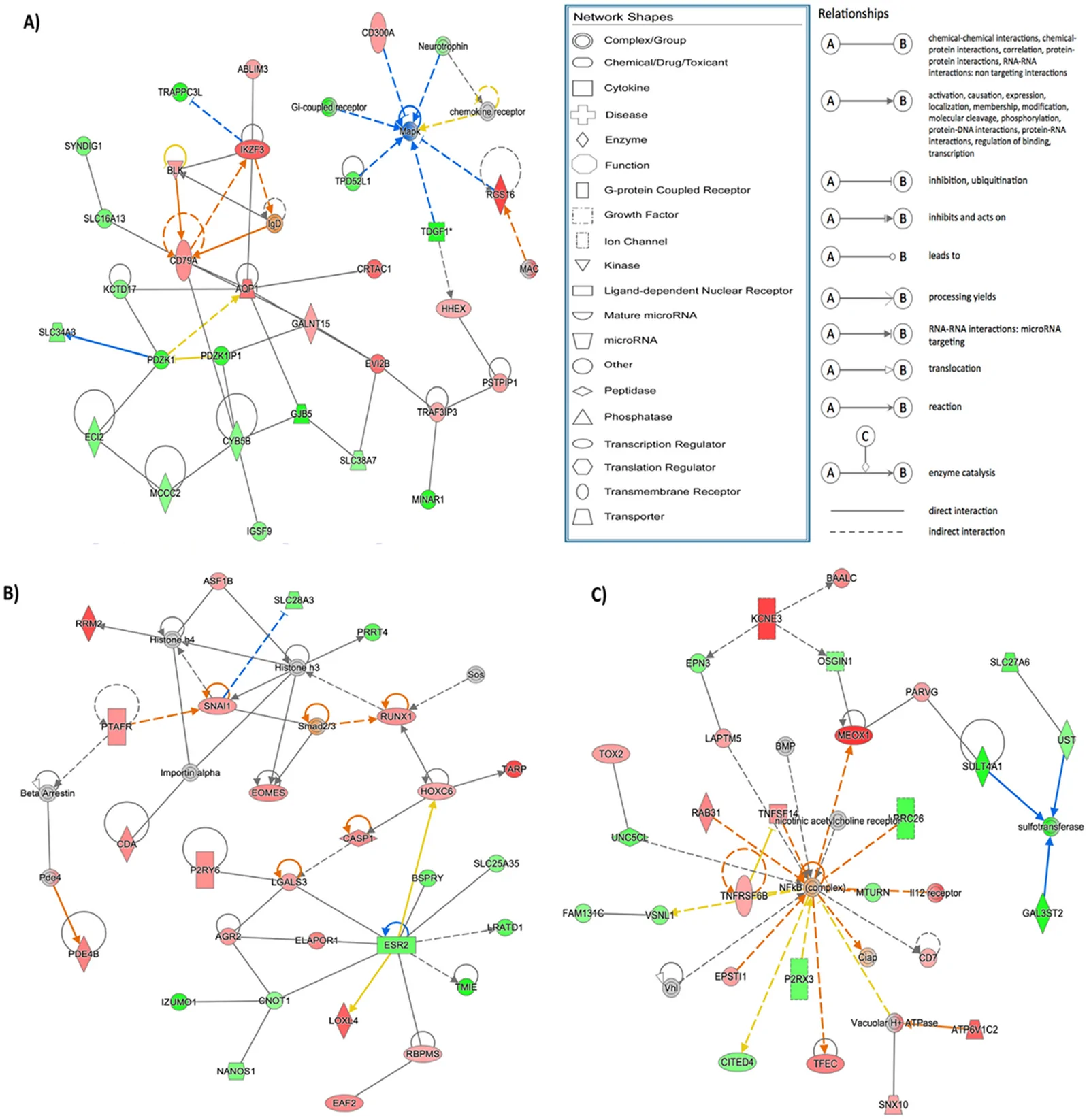
The top 3 IPA networks **(A-C)** illustrate the relationship and interaction between genes and molecules that regulate their functions in the bovine endometrium. The network is displayed graphically as nodes (molecules). The node color intensity indicates the expression of genes, with red representing upregulation and green, downregulation in the Infectious vs. the Normal endometrium.

### Gene expression profiles using qPCR in the control vs infectious bovine endometrium

Differential gene expression between infected and healthy uterine tracts was analyzed using RNA-Seq analysis. The most highly upregulated and downregulated genes were subsequently validated using qPCR. Among the upregulated candidate genes, *SERPINA14, CHI3L1, C15H11orf34, SLPI, BOLA-DQA5, CCL19, DMBT1, IGHG1, IGCGAMMA, LOC112443200, and LOC100300716*, the expression of *CHI3L1, SLPI* and *CCL19* were found to be significant (Figure 7). Among the downregulated candidate genes, *GSX1, CNGA1, TDGF1, HTR1A, BPIFA1, PIPOX, CBLN4, PRKCG, TMIE, ST8SIA6, SH2D4B, LY6L, LOC112444733*, and *LOC100847874*, the expression of *GSX1, TDGF1, BPIFA1, PIPOX, CBLN4, PRKCG, TMIE, ST8SIA6, LY6L, LOC100847874* were found to be significant (Figure 7). Interestingly, *CNGA1* showed a higher fold change in the infected uteri than the normal sample.

**Figure 7:**
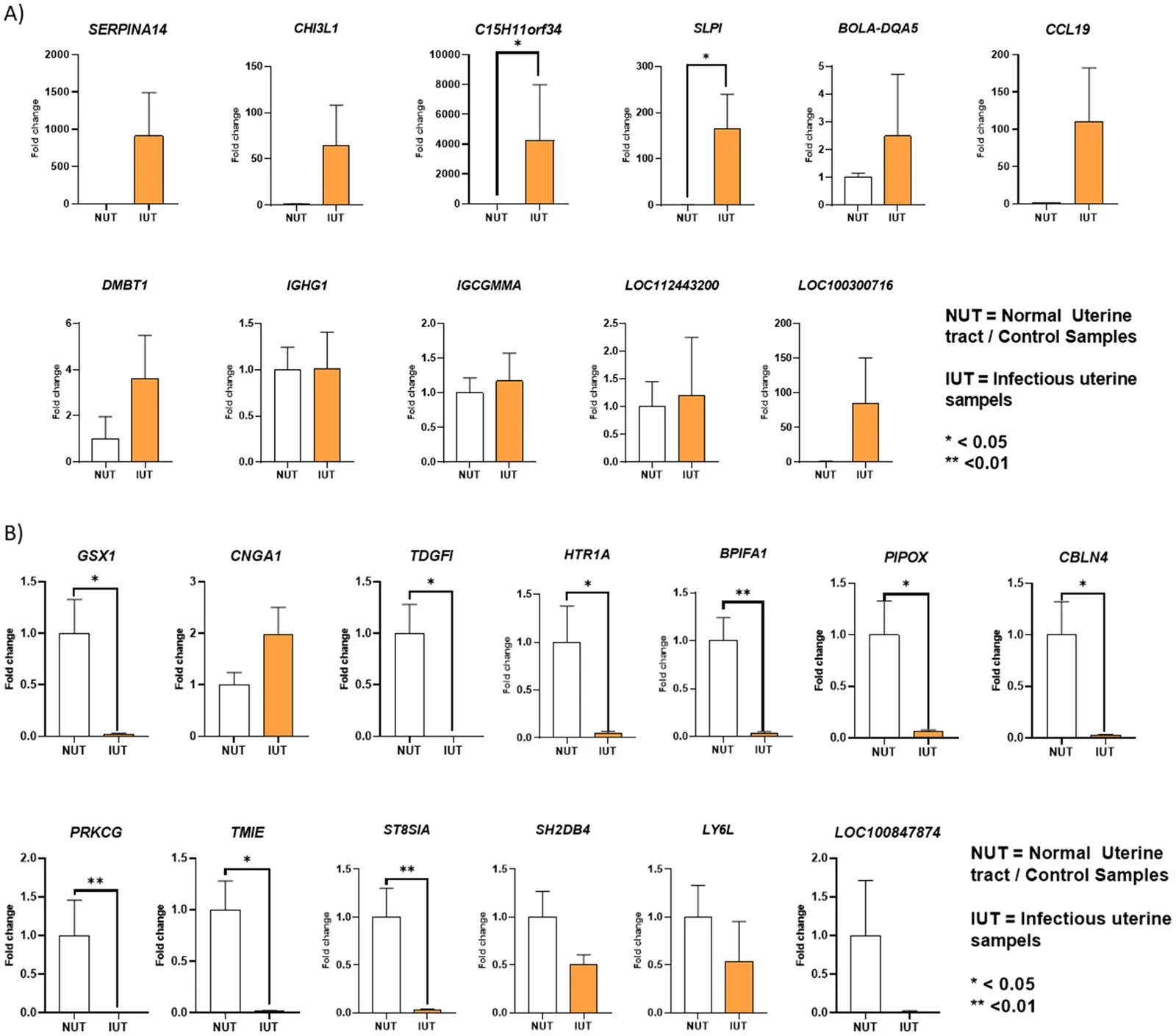
Validation of the upregulated **(A)** and downregulated **(B)** DEGs expressions in the endometrium. The fold changes were normalized with Beta-actin. Data represented as the mean ± standard error. The x-axis represents the different treatment groups of the cows (NUT, Normal Uterine Tract; IUT, Infectious Uterine Tract). Y-axis represents relative fold change for gene expression.

## Discussion

The uterus plays a crucial role in reproductive cyclicity and provides the biological environment for the establishment and maintenance of pregnancy. Uterine infections have a range of detrimental effects on reproductive health, including infertility, abortion, preterm labor, and clinical diseases (1, 17, 26–28). The effects of these infections can be difficult to detect, as their severity can vary from being undetectable to causing early slaughter or even death of the animals (1). After infection, the lining of the uterus can become filled with inflammatory cells like plasma cells, neutrophils, and lymphocytes, resulting in increased blood flow, congestion, shedding, and superficial necrosis of the endometrial layer (1, 29–31). The severity of the infection will determine the extent of changes in the uterus (1). Our study aimed to investigate the transcriptomic response of the intercaruncular endometrium in response to uterine infection, as it supplies essential nutrients and creates a suitable environment for embryo implantation and pregnancy success.

Innate immunity is essential for protecting the body from invading microbes, relying primarily on the skin and mucosa as physical barriers. Additionally, the immune system employs antimicrobial peptides, the complement system, and immune cells to detect and respond to bacteria and other harmful substances. The immune response is mediated by hematopoietic cells, including monocytes, macrophages, eosinophils, neutrophils, and natural killer cells. These cells work with non-hematopoietic cells such as epithelial, stroma, and endothelial cells to provide nonspecific defense against pathogens (32). The immune cells recognize pathogenic organisms through pattern recognition receptors, which include Toll-like receptors (TLRs), nucleotide oligomerization domain (NOD)-like receptors (NLRs), retinoic acid-inducible gene I (RIG-I)-like receptors (RLRs), and C-type lectin receptors (CLRs) (33). This recognition process allows for a rapid and effective response to potential threats. Our study showed an increase in the expression of several TLR receptors and related factors, including *TLR2, TLR3, TLR4, TLR5, TLR7, TLR8, TRAF6, MAPK3 (ERK1), MAPK14, MYD88, TRAM1, TRAM2, TIRAP*, and *LY96*. Out of these, *TLR4* and *TLR7* were significantly expressed in the bovine endometrium. TLR4 is crucial in identifying LPS in the endometrium and is the key regulator of the innate immune system's response to bacterial LPS in the reproductive tract (9). *TLR4*, in combination with *CD14* and *LY96* (*MD2*), recognizes gram-negative bacteria's lipopolysaccharide (17). In our study, we observed significant upregulation of *CD14* and increased expression of *LY96*. Further, *STAT3* was significantly upregulated, which plays a crucial role in TLR4-dependent production of *IL6* and *IL8* (34). Additionally, *TLR3, TLR7*, and *TLR8* are associated with recognizing nucleic acids, often from viruses (17). *MYD88* recruits *IRAK4*, which then activates both *TRAF6* and *IRAK1*, leading to the activation of the *IKK* complex. This facilitates the movement of the nuclear transcription factor (NFkB) from the cytoplasm to the nucleus (32). Our study revealed that the NF-κB signaling pathway and genes, such as NF-KB1 and NF-KB2, were significantly upregulated. *TRAF6* also stimulates the activation of MAPK (p38 and ERK1/2), which subsequently activates the AP1 transcription factor. The activation of both the NFkB and MAPK-dependent pathways results in the production of cytokines, chemokines, prostaglandins, and other inflammatory mediators (32). Nucleotide-binding oligomerization domain-like receptors (NLRs) are activated by pathogen-associated molecular patterns (PAMPs) or damage-associated molecules. These NLRs participate in cytokine production and autophagy following infection (32, 35). The present study identified increased expression of certain NLRs and NLR-associated genes, including *NOD1* (significantly upregulated), *CITTA, NAIP, NLRP1, NLRP3*, and *NLRP6*. NOD1 and NOD2 stimulate the production of pro-inflammatory cytokines and antimicrobial peptides by activating NFkB (32). The NLR family members, specifically NLRP1, NLRP3, and NLRC4, in conjunction with AIM2, trigger the activation of the inflammasome, leading to the activation of caspase-1. The activated Caspase-1 processes IL-1β, IL-18, and IL-33 precursors into their biologically active forms, which are subsequently secreted from cells (32, 36). Our study has shown that the levels of *NLRP1, NLRP3*, and *NLRC4* have significantly increased, along with *IL1*β, *IL18*, and *IL33*. It's worth noting that *CITTA* (NLRA) regulates the transcription of MHC class II antigens, and *NAIP* (*NLRB*) plays a crucial role in host defense and cell survival (37, 38). Within the RLR family, *DDX58* and *IFIH1*, encoding *RIG-I* and *MDA5*, demonstrated increased expression levels. Notably, DDX58 exhibited significant upregulation. RLRs perform the critical function of stimulating type 1 interferon production, playing an indispensable role in initiating antiviral responses (39). Furthermore, genes encoding various CLRs, including CLC7A (Dectin 1), CD209 (DC-SIGN), CLEC6A (an ortholog of the human Dectin 2 coding gene), and MRC1 (the mannose receptors), exhibited increased expression. CLRs activate the lectin complement pathway, promoting neutrophil chemotaxis (32). Our study findings show that when a uterine infection is present, pattern recognition receptors and their associated genes are upregulated and activated. This leads to an innate immune response, resulting in the production of inflammatory mediators such as cytokines, chemokines, and prostaglandins. This response is significant as it highlights the crucial role of the innate immune system in effectively combating uterine infections.

To combat microbial pathogens that invade the uterus, a swift and effective response is essential. Our study found that a group of pro-inflammatory genes, which includes *IL1A, IL1B, IL1R1, IL6, IL8* (*CXCL8*), *IL9, IL12A, IL12B, IL17A, CXCL5, CCL2, CCL3, CCL4, CSF2, CSF3, TNF*, IFNG, and prostaglandin E synthase (PTGES), exhibited increased expression. Among them, *IL6, IL12A, CXCL8, CCL2, CCL4, TNF*, and *PTGES* were significantly upregulated. These genes attract neutrophils and macrophages to help combat infections and resolve diseases. Their chemoattractants, such as *IL8, MCP1* (*CCL2*), and *MIP1B* (*CCL4*), draw neutrophils, monocytes, and T cells to the site of inflammation. The proinflammatory mediators further stimulate inflammation by attracting and stimulating immune cells. TNF-alpha, IL6, GM-CSF (CSF2), and G-CSF (CSF3) upregulate immune cells, which secrete more of these and other proinflammatory cytokines. This initiates leukocyte differentiation into more functionally active cells (28). The presence of Interferon Gamma (IFNG) has been shown to induce the production of nitric oxide, which in turn stimulates the release of tumor necrosis factor (TNF). In synergy with Interleukin 1 (IL1), this combined effect triggers a cascade of inflammation that leads to tissue destruction and a loss of function. Conversely, anti-inflammatory genes such as Interleukin 4 (IL4), Transforming Growth Factor Beta 1 (TGFB1), TGFB2, and TGFB3 are responsible for suppressing the production of IL1, TNF, IL8, and vascular adhesion molecules (40). This study reveals that *TGFB1* expression was significantly increased, while that of *IL13* was decreased. It is important to note that the balance between the pro-inflammatory and anti-inflammatory genes determines the outcome of the disease (40). Furthermore, our findings show the activation of several proinflammatory genes that regulate different immune cells to act against invading pathogens and promote recovery from the infection. In addition to the immune response, the body's ancient component, antimicrobial peptides (AMPs), provides an additional defense mechanism against pathogens (41, 42). Our study found that several AMPs, including lingual AMP (LAP), tracheal AMP (TAP), and members of the defensin beta family (*DEFB1, DEFB4A, DEFB7, DEFB10*, and *DEFB13*), showed an increased expression. These AMPs can directly kill the pathogen or modulate host defense against the pathogen.

The galectin family can potentially regulate uterine immunity by targeting the glycoproteins with multiple galactose-β1,4-N-acetylglucosamine units on their cell surface. Our research suggests that the expression of galectin 1 (*LGALS1*) has increased, while galectin 3 (*LGALS3*) has been significantly upregulated. Galectin 1 can control cell proliferation, effector T cells, neutrophil survival, and extravasation *in vivo*. On the other hand, galectin 3 regulates the adherence of T cells to endothelial cells and dendritic cells or macrophages (43–46).

Infertility resulting from uterine infection may be due to uncontrolled and excessive endometrial response to the infection, as indicated by numerous studies (5). In our study, we observed a significant increase in the expression of various proinflammatory genes, including *IL1A, IL1B, IL17A, IL6, IL9 TNF, CXCL5*, and *PTGES*. Another study on cows reported higher expression of IL1A, IL1B, and IL6 in infertile animals, suggesting that our study animals might develop infertility after the infection (47).

Prostaglandin (PGE) secretions from endometrial cells are associated with fertility, as PGE plays multiple roles in regulating endometrial function and luteolysis. PGE is also important for controlling inflammation through PTGER2 and PTGER4 in a context-dependent manner (17, 48, 49). Our study found that the expression of *PTGER4* was significantly decreased, while the expression of *PTGER2* increased. This is similar to findings in infertile animals (47).

Our study found that the inflammatory response in animals led to the activation of several functions, including migration, chemotaxis, and activation of various immune cells such as leukocytes, phagocytes, neutrophils, dendritic cells, mononuclear leukocytes, antigen-presenting cells, and T cells. This response also led to an infiltration of these types of cells. The analysis also predicted an increase in the quantity of several cells, including T lymphocytes, B lymphocytes, plasma cells, thymocytes, double-positive thymocytes, lymphatic system cells, CD4+ T-lymphocytes, and helper T lymphocytes. The activation of several master regulators, such as transmembrane receptors, cytokines, transmembrane regulators, and peptidase, was also identified through the analysis. Upstream analysis revealed that *LY96, IFNG, IRF4, CD14, IL1B, CCL15, MAP3K, MMP17, MMP2, TLR7/8, TRAF, IL2, STAT3, IL6, IL21, IL1A, IL15, IL12* (complex), *IL5, IL12* (family), *IL7, CLEC7A*, and *CLEC12A* are among the activated regulators.

This study has a few limitations. First, we did not confirm the specific infectious organisms, as our primary objective was to characterize infection-associated transcriptomic changes rather than identify causative pathogens. Future studies should incorporate pathogen-specific diagnostic approaches to establish etiology. Second, the relatively small sample size was constrained by the availability of samples from external facilities; therefore, we followed previously published transcriptomic studies using at least five biological replicates per group. Finally, we based IPA networks largely on human datasets because bovine-specific information is limited. Although this may limit species-specific interpretation, the analysis provides valuable insights into regulatory pathways potentially affected by infection in the bovine endometrium.

In conclusion, our study revealed a clear enrichment of several components of the innate immune response, including TLR receptors, NLR receptors, and RLR receptors, which are involved in recognizing invading pathogens. Furthermore, we identified an enrichment of cytokines and chemokines, which play a vital role in the pro-inflammatory and anti-inflammatory response against invading pathogens. Our IPA analysis of the study reveals several master regulators that influence the actions of several molecules. The study found that several biological processes and pathways related to the immune response were enriched in response to the invading pathogens causing uterine infections. Our research showed that the immune system is activated to fight the pathogens causing the infection, with the most enriched biological processes being inflammatory and immune response. The KEGG pathway analysis identified cytokine-cytokine receptor interaction and chemokine signaling, indicating that the immune system works to neutralize the pathogen and restore the body to normal conditions. As a result, non-specific uterine infections can cause significant changes in the physiology and environment, leading to infertility and economic loss for ranchers.

## Data Availability

The datasets used and/or analyzed during the current study are available from the corresponding author upon reasonable request. RNA-sequencing data were submitted to the NCBI GEO repository (GSE209700): https://www.ncbi.nlm.nih.gov/geo/query/acc.cgi?acc=GSE209700.
